# Detection of a substantial number of sub-microscopic *Plasmodium falciparum* infections by polymerase chain reaction: a potential threat to malaria control and diagnosis in Ethiopia

**DOI:** 10.1186/1475-2875-12-352

**Published:** 2013-10-03

**Authors:** Lemu Golassa, Nizar Enweji, Berhanu Erko, Abraham Aseffa, Göte Swedberg

**Affiliations:** 1 Aklilu Lemma Institute of Pathobiology, Addis Ababa University, Addis Ababa, Ethiopia; 2 Armauer Hansen Research Institute, Addis Ababa, Ethiopia; 3 Medical Biochemistry and Microbiology, Uppsala University, Uppsala, Sweden

**Keywords:** Sub-microscopic carriage, Asymptomatic malaria, Microscopy, RDT, PCR, Ethiopia

## Abstract

**Background:**

Prompt and effective malaria diagnosis not only alleviates individual suffering, but also decreases malaria transmission at the community level. The commonly used diagnostic methods, microscopy and rapid diagnostic tests, are usually insensitive at very low-density parasitaemia. Molecular techniques, on the other hand, allow the detection of low-level, sub-microscopic parasitaemia. This study aimed to explore the presence of sub-microscopic *Plasmodium falciparum* infections using polymerase chain reaction (PCR). The PCR-based parasite prevalence was compared against microscopy and rapid diagnostic test (RDT).

**Methods:**

This study used 1,453 blood samples collected from clinical patients and sub-clinical subjects to determine the prevalence of sub-microscopic *P. falciparum* carriages. Subsets of RDT and microscopy negative blood samples were tested by PCR while all RDT and microscopically confirmed *P. falciparum*-infected samples were subjected to PCR. Finger-prick blood samples spotted on filter paper were used for parasite genomic DNA extraction.

**Results:**

The prevalence of sub-microscopic *P. falciparum* carriage was 19.2% (77/400) (95% CI = 15. 4–23.1). Microscopy-based prevalence of *P. falciparum* infection was 3.7% (54/1,453) while the prevalence was 6.9% (100/1,453) using RDT alone. Using microscopy and PCR, the estimated parasite prevalence was 20.6% if PCR were performed in 1,453 blood samples. The prevalence was estimated to be 22.7% if RDT and PCR were used. Of 54 microscopically confirmed *P. falciparum*-infected subjects, PCR detected 90.7% (49/54). Out of 100 RDT-confirmed *P. falciparum* infections; PCR detected 80.0% (80/100). The sensitivity of PCR relative to microscopy and RDT was, therefore, 90.7% and 80%, respectively. The sensitivity of microscopy and RDT relative to PCR was 16.5 (49/299) and 24.2% (80/330), respectively. The overall PCR-based prevalence of *P. falciparum* infection was 5.6- and 3.3 fold higher than that determined by microscopy and RDT, respectively. None of the sub-microscopic subjects had severe anaemia, though 29.4% had mild anaemia (10–11.9 g/dl).

**Conclusions:**

Asymptomatic, low-density malaria infection was common in the study area and PCR may be a better tool for measuring *Plasmodium* prevalence than microscopy and RDT. The inadequate sensitivity of the diagnostic methods to detect substantial number of sub-microscopic parasitaemia would undoubtedly affect malaria control efforts, making reduction of transmission more difficult. RDT and microscopy-based prevalence studies and subsequent reports of reduction in malaria incidence underestimate the true pictures of *P. falciparum* infections in the community. PCR, on the other hand, seems to have reasonable sensitivity to detect a higher number of infected subjects with low and sub-microscopic parasite densities than RDTs or microscopy.

## Background

Individuals in malaria-endemic areas can carry microscopically detectable levels of *P. falciparum* asymptomatically and also carry sub-microscopic asymptomatic infections below the microscopic detection threshold that can only be detected using molecular techniques. Routinely used laboratory methods appropriate for large-scale use, such as microscopy and rapid diagnostic tests (RDTs), are not sensitive enough to detect low-grade, asymptomatic infections [1]. One of the greatest disadvantages of the microscopic diagnosis is the possibility of misdiagnosis of *Plasmodium* species, particularly for low parasitaemia [2]. Usually test sensitivity suffers when parasite densities within individual infections are low [3]. Since microscopy and RDT have limitations, low-density infections are likely missed during screening of endemic populations [4]. Polymerase chain reaction (PCR) is more sensitive than microscopy and RDT, and has been widely used for diagnosis, confirmation of diagnosis, epidemiological studies and drug efficacy assessment [5].

The contribution of the sub-microscopic reservoir to sustaining malaria transmission depends on malaria endemicity and slide positivity rate of a given area. Studies have shown that sub-microscopic carriers are presumed to be the source of over 20% of mosquito infections in areas where slide prevalence is less than 4% [6] while others suggested the contribution to be over 20% where slide prevalence is up to 24% and can be as high as 50% of mosquito infections in very low-transmission areas (slide prevalence <0.5%) [7]. Sub-microscopic infections are more important contributors to transmission in areas with low or very low transmission intensity (under ~0.5%) than to sustain transmission in areas of high transmission intensity. According to Okell and her colleagues [7] meta-analysis, sub-microscopic infections are important in sustaining transmission in areas where slide prevalence is low (<10 – 20%). More importantly, sub-microscopic carriers will become increasingly important as current control programmes continue to successfully reduce transmission intensity. Okell and her colleagues [7] have developed a simple model to estimate the prevalence of sub-microscopic carriage when PCR or slide prevalence is known (i.e. PCR prevalence - slide prevalence).

Prompt and effective malaria diagnosis not only alleviates suffering, but also decreases malaria transmission at the community level. Although microscopy remains the gold standard for malaria diagnosis, the detection threshold in Giemsa-stained thick blood film has been estimated to be 4 –20 parasites/μl [8]. Nonetheless, false positive results can be associated with poor blood film preparation that generates artifacts, including bacteria, fungi, stain precipitation, and dirt and cell debris all of which may be mistaken for malaria parasites [9]. On the other hand, the chance of false negative results increases with decreasing parasite densities. Improving diagnostic accuracy in malaria control systems can be both technically and financially challenging [10]. On the other hand, the sensitivity of RDT varies with *Plasmodium* species and parasitaemia [11]. As malaria transmission declines and countries progress towards malaria elimination the need to detect sub-microscopic infections is becoming increasingly important, since low-density infections among symptomatic and asymptomatic persons is likely to increase, which may limit the utility of RDTs [12].

In Ethiopia, microscopy and RDT are commonly used for malaria diagnosis although the diagnostic performances of these tests haven’t been evaluated against PCR. A PCR- based study conducted in low-transmission settings has demonstrated that a high proportion of cases with low-density parasitaemia were not detected by microscopy or RDT [5,13]. Studies from several malaria-endemic countries indicate that the proportion of low-density (<200 parasites/μl) infections in symptomatic persons is higher in low-transmission than in high-transmission areas, and also higher in *Plasmodium vivax* than in *P. falciparum* infections [9]. This suggests that a larger proportion of symptomatic cases may be missed in low-transmission settings noticeable in Ethiopia. Malaria control programmes will also need to actively monitor the sensitivity of RDTs and microscopy in detecting low-density parasitaemia in symptomatic patients presenting to health facilities and in population-based surveys, to capture asymptomatic infections with a more sensitive diagnostic method [12]. The objective of this study was, therefore, to determine the prevalence of sub-microscopic malaria carriage in blood samples collected from clinical patients and sub-clinical subjects.

## Methods

### Study site and sample collection

**Figure 1:**
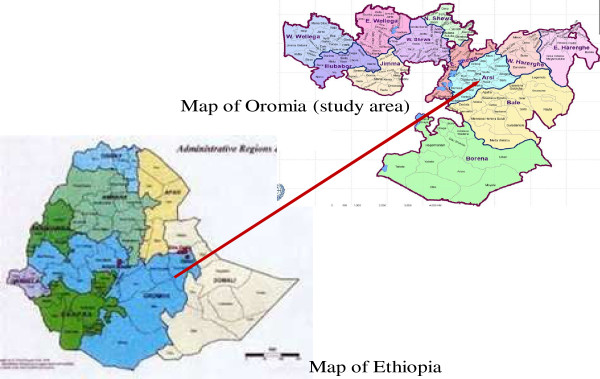
Location of study site.

**Figure 2:**
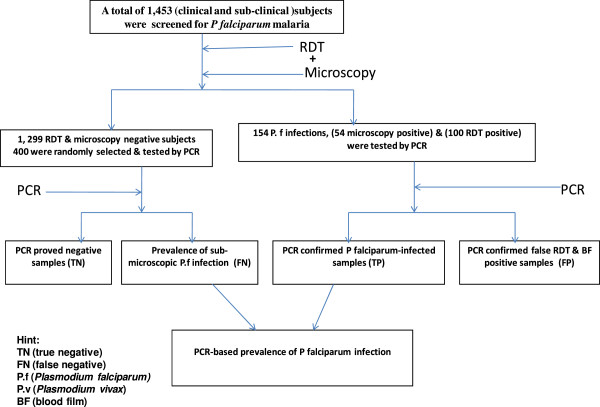
Study flow chart.

This study was conducted in West Arsi Zone, Shalla district, which is approximately 251 km from the capital, Addis Ababa, Ethiopia (Figure 1). The capital of the district, Aje town, is located at 0382146.3 E, 071734.2 N and 1,852 m above sea level. West Arsi zone has ten districts of which Shalla district has the highest number of malaria cases. *Plasmodium falciparum* and *P. vivax* are the two common causes of malaria. A cross-sectional study was conducted in 12 randomly selected *kebeles* (the smallest administrative unit) to determine the prevalence of asymptomatic malaria carriages from November through December 2012. The *kebeles* have known population size and systematically registered households. After obtaining informed consent from parents/guardians, members of the randomly selected households who met the inclusion criteria (resident of the *kebele* for at least 1 year, age greater or equal to two years, with no known acute &/or chronic illness, no history of fever in the last 72 hours, an axillary temperature <37.5°C, with no history of anti-malarial drug treatment within the last two weeks, absence of any malaria-related symptoms and willing to participate in the study) were requested to give finger-prick blood samples. Blood films were also collected from febrile patients attending three health centres to determine the prevalence of symptomatic malaria. Finger-prick blood was spotted on Whatman 3MM filter papers, air dried and individually kept in zip-lock plastic bag with desiccant for PCR. A total of 1,453 blood samples (1,094 blood samples from sub-clinical subjects and 359 blood samples from clinical patients) were collected. Four hundred samples were randomly selected from RDT- and microscopy-negative samples and tested by PCR to determine the prevalence of sub-microscopic *P. falciparum* infection. All RDT- and microscopy-positive samples from both clinical patients and sub-clinical subjects were tested by PCR to determine the diagnostic performances of the two tests (Figure 2).

### Blood film examination and determination of parasitaemia

Two independent, experienced microscopists, who were blinded to the patients’ clinical status and to the results of the RDTs, examined all coded smears for parasites. Identification of malaria species was done using the thin blood smear. A negative result was recorded after thorough examination of 25 fields (~200 leucocytes) without any parasite. For quantification of malaria parasites in the thick film, a total of 200 WBC were examined while simultaneously counting the number of asexual forms of malaria parasites. Plasmodial density was recorded as the number of parasites/200 WBC. This was done using a standard mean white blood cell count of 8,000 leukocytes μl^-1^[14]. Densities were calculated and converted into the number of parasites μl^-1^ of blood. RDTs were used in the survey to offer instant treatment to individuals with a positive test according to the national malaria diagnosis and treatment guideline.

### Rapid diagnostic tests (RDTs)

SD BIOLINE Malaria Ag P.f/P.v POCT test kits (Standard Diagnostic, Inc, Germany, LOT No: 145021) were used as per the manufacturer’s instructions. Finger-prick blood was used for the rapid assay. The kit is a one step, rapid, qualitative test intended for the detection of malaria infection in human blood samples indicating differential diagnosis between P. f HRP-II (*P. falciparum* specific histidine-rich protein-II) and pLDH (*Plasmodium* lactate dehydrogenase) specific to *P. vivax*. A blood sample was considered positive for falciparum malaria if two colour bands (*P. falciparum* test line and control line) appeared within the result window. On the other hand, the sample was considered positive for vivax malaria if two colour bands (*P. vivax* test line and control line) appeared within the result window. If three colour bands (*P. falciparum* test line, *P. vivax* test line and control line) appeared within the result window, it was considered mixed infection (*P. falciparum* and *P. vivax)*. The presence of only one colour band (control line) was considered negative but if the control band failed to appear within the result window, it was considered invalid.

### DNA extraction and polymerase chain reaction (PCR)

Parasites DNA was extracted from the blood spotted on Whatman 3MM filter paper samples. A 3-mm^2^ square piece of blood-impregnated filter paper (~15 μl) was excised using a puncher and transferred in to a 1.5 μl tube and stored at −20°C until use. The extraction was done using the Chelex with heat extraction method [15]. Primary and nested PCR assays were performed in a 20 μl volume reaction mixture with 2 μl dream Taq buffer, 100 μM dNTPs, 0.05 μM μl each primers and 1.25 μM dream Taq enzyme. Two microliters (2 μl) of the sample DNA were used for outer amplification. For nested amplification, 2 μl of the outer PCR products was used as a template DNA. The extracted DNA was amplified by nested PCR (LifePro thermal cycler Bioer). A negative and two positive controls (D2 and 3D7) were consistently used for the quality control. DNA extraction was done at Uppsala University, Uppsala, Sweden. The thermocycling conditions for the outer PCR were: initial activation at 94°C for 3 min, followed by 44 cycles of 94°C for 30 seconds, 56°C for 30 seconds, 60°C for 1 min and finally 60°C for 3 min. For the nested PCR: 95°C for 5 min, followed by 30 cycles of 92°C for 30 sec, 48°C for 30 seconds, and 65°C for 30 seconds and finally 65°C for 3 min. Five microliters (5 μl) of the nested products were analysed using 1.5% agarose gels. The PCR products were then visualized by ethidium bromide staining under UV light.

### Ethics consideration

This protocol was approved by the Institutional Review Boards (IRBs) of Aklilu Lemma Institute of Pathobiology, Addis Ababa University and of the Armauer Hansen Research Institute as well as the National Research Ethics Review Committee (NRERC). Information about the objective of the study was given to the head of the household or study subjects. Verbal informed consent was sought from each eligible subject and parents of children < six years of age for blood films. Additional verbal informed assent was sought from children aged six to18 years. Illiterate people signed with their fingerprint. The privacy of participants was preserved by allocating codes/numbers to participants in the blood survey instead of names. Study subjects who tested positive for malaria by RDT were treated according to the national malaria treatment guideline in the field.

### Statistical analysis

PCR-based prevalence of sub-microscopic *P. falciparum* carriage was analysed across age groups to determine age-related correlation. Statistical analysis was performed using Stata version 11. The association between haemoglobin level and sub-microscopic carriages were determined. The sensitivity and specificity of RDT and microscopy was determined taking PCR as the ‘reference’. The X^**2**^ test was used to compare the *Plasmodium* carriage among different age groups. Logistic regression was made to determine the association of parasite prevalence by PCR and the different independent variables. A test was considered statistically significant if the p-value was < 0.05 (Table 1).

**Table 1:** **Primer sequences for detection of *Plasmodium falciparum *DNA**

**Target name**	**PCR**	**Sequence (5’-3’)**
CRTP1	Outer Forward	CCGTTAATAATAAATACACGCAG
CRTP2	Outer Reverse	CGGATGTTACAAAACTATAGTTACC
CRTD1	Nested Forward	TGTGCTCATGTGTTTAAACTT
CRTD2	Nested Reverse	CAAAACTATAGTTACCAATTTTG

## Results

### Prevalence of sub-microscopic carriage as determined by polymerase chain reaction

After excluding 154 samples that were positive by microscopy and/or RDT, 400 negative samples from a total of 1, 299 were randomly selected and tested by PCR. The prevalence of sub-microscopic *P. falciparum* carriages (carriers were defined as those individuals with infections detected by PCR but not by microscopy or RDT) was 19.2% (77/400). Although sub-microscopic infections was higher in males (22.5%) than females (16.6%), there was no statistically significant difference (X^2^ = 2.20; P > 0.05) in sub-microscopic infections between sexes. The mean haemoglobin value observed in 172 study subjects was 16.5 g/dl (95% CI = 15.9-17.3). Anaemia (<12 g/dl) was observed in 21 (12.2%) of the study subjects (Table 2). There was no statistically significant association between sub-microscopic *P. falciparum* carriages and haemoglobin level (X^2^ = 0.47, P > 0. 05). Among five individuals with body temperature ≥ 37.5°C, 20.0% (one of five) had *P. falciparum* infections while 19.2% (76/395) individuals with body temperature < 37.5°C had infections.

**Table 2:** Characteristics of the study population and sub-microscopic carriages

**Characteristics**	**# examined**	**Sub-microscopic carriers as determined by PCR, +ve (%)**
**Sex (n = 400)**		
Male	178	40 (22.5)
Female	222	37 (16.7)
**Age in years (n = 400)**		
2-5	49	5 (10.2 )
6-15	144	29 (20.1)
16-25	77	16 (20.8)
26-35	88	20 (22.7)
>35	42	7 (16.7)
**Body temp (n = 400)**		
<37.5	394	76 (19.3)
≥ 37.5	6	1 (16.7)
**Hb level (g/dl) (n = 172)**		
Normal (> = 12 g/dl)	151	27 (17.9)
Mild (10–11.9 g/dl)	17	5 (29.4)
Moderate (7–9.9 g/dl)	3	0 (0)
Severe (<7 g/dl)	1	0 (0)

Anaemia haemoglobin (Hb) was defined as normal (>12 g/dl), mild (10–11.9 g/dl), moderate (7–9.9 g/dl) and severe (<7 g/dl) anaemia.

### Polymerase chain reaction-based prevalence of *Plasmodium falciparum* infection

The prevalence of *P. falciparum* infection was 3.7% (95% CI, 2.7-4.7) (54/1,453) and 6.9% (95% CI, 5.5-8.1) (100/1,453) as diagnosed by microscopy and RDT, respectively (Table 3). Of the microscopy-positive samples, PCR detected 90.7% (49/54) while it detected 80% (80/100) from RDT-positive samples. To calculate the overall PCR-based parasite prevalence, given the PCR positivity rate of 19.2% (77/400) applies to the entire RDT- and microscopy-negative samples, 250 samples would be expected to be PCR-positive from a total of 1, 299 samples. By adding up 49 samples (including mixed infection) positive by both microscopy and PCR (Table 3), the total number of PCR positive samples in 1,453 samples would be 299, giving an overall parasite prevalence of 20.6%, 5.6-fold higher than that determined by microscopy alone. On the other hand, by adding 80 samples (including mixed infection) positive by both RDT and PCR, the total number of PCR-positive samples in 1,453 samples would be 330, giving an overall parasite prevalence of 22.7%, 3.3-fold higher than that determined by RDT only (Table 2). The estimated parasite prevalence using PCR and microscopy was approximately 20.6% (299/1,453) from the total samples. This results show that microscopy and RDT had a substantially low sensitivity in detecting sub-clinical parasitaemia. Thus, the sensitivity of microscopy relative to the PCR was 16.4% which was calculated as % positive by both microscopy and PCR / % positive by PCR (49/1,453)/299/1,453). The sensitivity of RDT relative to the PCR was 24.2% and calculated as % positive by both RDT and PCR / % positive by PCR (80**/**1,453**/**330**/**1,453). Moreover, the high number of false negatives by microscopy and false positives by RDT raises serious doubts about their effectiveness as the sole screening tests especially in places such as this study area. Equally important are also a high number of false negatives by RDT as well as false positives.

**Table 3:** Evaluation of rapid diagnostic test- and microscopy-positive results against polymerase chain reaction

	Microscopy (n = 54)*	RDT (n = 100)*		
**PCR**	***P. falciparum***	**Mixed (Pf/Pv)**	***P. falciparum***	**Mixed (Pf/Pv)**
*P. falciparum*	44	5	73	7
Negative	3	2	20	0
Total	47	7	93	7

*Only microscopy-and *RDT*-positive samples were subjected to *PCR* (the overall results of microscopy and *RDT* survey aren’t indicated in the table).

### Comparison of microscopy, rapid diagnostic test and polymerase chain reaction for detection of *Plasmodium falciparum* infection

PCR consistently detected higher frequencies of *P. falciparum* infection than microscopy and RDT. A total of 54 blood films and 100 RDT-confirmed *P. falciparum*-infected samples were separately tested by PCR. Of the 54 microscopy-positive samples, PCR detected *P. falciparum* DNA in 49 of the samples. Thus, the specificity of the microscopy was 90.7%. On the other hand, of 100 RDT-positive samples, PCR detected *P. falciparum* DNA in 80 of the samples. RDT showed 80.0% specificity for *P. falciparum* detection (Table 3). The prevalence of false positive was 9.3 and 20.0% for microscopy and RDT, respectively. PCR had statistically significant association with RDT (X^2^ = 53.7, P = 0.00) and microscopy (X^2^ = 48.1, P = 0.00) in positive samples. Microscopy and RDT detected on average, 38.9% (49/126) and 60.0% (80/157) of all PCR-detected *P. falciparum* infections, respectively.

### Rapid diagnostic test- and microscopy-based prevalence of microscopic and sub-microscopic *Plasmodium falciparum* by age group

There was statistical significant association between age groups and RDT (X^2^ = 63; P < 0.000) and microscopy (X^2^ = 46; P = 0.000) positivity rate of microscopic *P. falciparum* infections, but there was no significant association between age and sub-microscopic carriages (p > 0.05). Microscopic *P. falciparum* prevalence ranged from as low as 1.9% (>35 years) to as high as 7.0% (two of five years) as determined by microscopy while it ranged from 1.9% (>35 years) to 12.6% (two of five years) using RDT. The prevalence of sub-microscopic *P. falciparum* infection ranged from as low as 10.2% (two of five years) to as high as 22.7% (26–35 years) (Table 4). PCR showed an unequivocal superior diagnostic performance compared to both RDT and microscopy. In this study, the proportion of positive test results for microscopic *P. falciparum* subjects in age two to five years increased from 7.0 to 12.6% as diagnosed by microscopy and RDT, respectively (Table 4). RDT was invariably more sensitive than blood film in detecting *P. falciparum* infections across all age groups.

**Table 4:** **Prevalence of microscopic and sub-microscopic *Plasmodium falciparum *by age group as diagnosed by rapid diagnostic test, blood film (microscopy) and polymerase chain reaction**

	**Prevalence of microscopic infections (P. f and mixed), n = 1, 453**		**Prevalence of sub-microscopic infections (P. f & mixed), n = 400**
**Age group, years**	**Microscopy,% (N)**	**RDT,% (N)**	PCR*,% (N)
2-5	7.0 (15/214)	12.6 (27/214)	10.2 (5/49)
6-15	3.9 (19/485)	9.1 (44/485)	20.1 (29/144)
16-25	2.7 (9/331)	5.4 (18/331)	20.8 (16/77)
26-35	4.1 (11/268)	4.9 (13/268)	22.7 (20/88)
>35	1.9 (3/155)	1.9 (3/155)	16.7 (7/42)

*are *PCR*- positive samples that were negative by both microscopy and *RDT* and it does not include *PCR* positives that were also positive by microscopy or *RDT*.

### Risk factors associated with sub-microscopic carriages

Surprisingly, the use of bed nets was not found protective against sub-microscopic carriage in both univariate analysis (OR = 0.39; p = 0.002) and multivariate analysis (OR = 0.40; p = 0.003). In this study, the overall self-reported bed net use was 62.3% (38.2% in men and 57.4% in women). Although sub-microscopic parasite carriages were high in older age groups, the difference was not statistically significant in both univariate and multivariate analysis. Sub-microscopic carriage was not significantly different between sexes (OR = 0.69; p = 0.145) in univariate logistic regression. Asymptomatic carriages were lowest among subjects with body temperature < 37.5°C although statistically not significant. Although sub-microscopic parasite carriage was higher among anaemic subjects than those with normal haemoglobin level, the difference was not statistically significant (Table 5).

**Table 5:** **Risk factors analysis for sub-microscopic *Plasmodium falciparum *carriage**

		**Univariate analysis**		**Multivariate analysis**	
**Covariates**	**# positive (N)**	**OR (95% CI)**	***p-value***	**OR (95% CI)**	***p-value***
**Age group, years**					
<5	5 (49)	1.00	-	1.00	-
6-15	29 (144)	2.21 (0.80-6.09)	0.122	1.58 (0.37-6.71)	0.530
16-25	16 (77)	2.30 (0.78-6.77)	0.128	0.89 (0.14-5.56)	0.906
26-35	20 (88)	2.58 (0.90-7.40)	0.076	2.04 (0.38-10.84)	0.399
>35	7 (42)	1.76 (0.51-6.02)	0.368	1.86 (0.30-11.46)	0.503
**Sex**					
Male	40 (178)	1.00	-	1.00	-
Female	37 (222)	0.69 (0.41-1.13)	0.145	1.29 (0.55-3.02)	0.553
**Bed net use**					
Yes	60 (249)	1.00	-	1.00	-
No	17 (151)	0.39 (0.22-0.71)	0.002	0.40 (0.22-.072)	0.003
**Fever**					
Yes (*≥*37.5)	1 (6)	0.83 (0.09-7.26)	0.872		
No (<37.5)	76 (394)	1.00	-		
**Hb level (g/dl)**					
Anaemic	5 (21)	1.00	-	1.00	-
Normal	27 (151)	0.69 (0.23-2.06)	0.515	1.09 (0.83-1.44)	0.503

*OR* (Odds ratio), *CI* (Confidence interval), *Hb* (haemoglobin) level: Normal >12 g/dl, Anaemic <11.9 g/dl.

## Discussion

The PCR analyses done here helped to establish two relationships, i.e. the rate of false positives and false negatives given by microscopy and RDT, respectively. Both have implications for malaria control measures. Starting with the false positive rate and its implications, in the present study, anti-malarial drugs were prescribed for 9.3 and 20.0% microscopy- and RDT-based parasite-negative patients, respectively, as witnessed by PCR. This percentage of parasite-negative patients receiving anti-malarial drugs was comparable to countries such as Tanzania [16], Uganda [17] and Zanzibar [18] that have high malaria transmission settings. The percentage of false positive patients (parasite-negative patients) receiving anti-malarials in the study under report was much lower than patients from low-moderate malaria transmission areas in Tanzania (63.0%) [19]. In this study, in five microscopy-positive samples (three *P. falciparum* and two mixed infections) all of which were determined as *P. falciparum*, PCR failed to detect them. They were considered false microscopy positives since PCR was considered the reference. Of the RDT positives, PCR failed to detect 20 samples, all of which were determined as *P. falciparum*, by RDT and hence called false RDT positives. Among many other factors, the utility of a RDT-based negative result depends on the sensitivity of the test [20], setting [21], brand [22], storage conditions (>30°C), and low levels of parasitaemia [4,23].

For false negatives, the discussion spins around 19.2% prevalence of sub-microscopic *P. falciparum*. The results of this study indicate that considerable numbers of *P. falciparum* infections were missed by both RDT and microscopy, suggesting the need for a more sensitive assay for the detection of sub-microscopic parasitaemia. There was no difference in sub-microscopic carriages in febrile and non-febrile patients (OR = 0.83; P = 0.872). The identification of sub-microscopic *P. falciparum* infection in fever patients may not help to reliably confirm malaria as the cause of the fever and excludes the possibility of other diseases [23]. The presence of sub-microscopic asymptomatic *P. falciparum* infections may represent a significant challenge to malaria control programmes since such parasitaemic individuals may serve as a reservoir of infection and contribute to mosquito infection [24-26]. Moreover, a mass screening and treatment campaign may include such individuals, but their parasitaemia would remain invisible to the classical light microscopy or RDT and this necessitates the use of molecular screening techniques. Studies have indicated that microscopy misses on average half of all *P. falciparum* infections in endemic areas compared to PCR [27]. The agreement between RDT and microscopy was 89.4% (42/47) and 71.4% (five of seven) in detecting *P. falciparum* and mixed infections, respectively. Both RDT and microscopy underestimated the true parasite prevalence in the study area. In this study, microscopy and RDT detected 38.9 and 60% of the infection identified by PCR.

The limitations of commercially available RDT kits are well documented [27,28]. The false positivity in RDT may arise due to circulating PfHRP2 antigen from recent infections [5,29]. Because RDTs that detect HRP-2 antigen cannot distinguish between active infections and resolved infections. RDTs based on detection of the HRP-2 antigen often remain positive for over five weeks after the disappearance of live parasites, because they detect the HRP-2 antigen which is still present in debris from dead parasites for some time after total parasite clearance [30]. In this study, false positivity rate of RDT was 2.1% higher than microscopy. The false positive rate of RDT in this study was fairly in agreement with previous evaluations of RDTs in population-based household surveys among healthy persons in Ethiopia [8], 1.5%, but much lower than reported in Zambia [31], 7.9%, when compared with microscopy. Such false positive readings in malaria diagnostic tests will overestimate the true parasite prevalence compared with expert microscopy. In spite of the apparent low sensitivity of RDT compared to PCR in this study, whether any deletion of HRP2-antigen exists among *P. falciparum* in Ethiopia requires further research.

The identification of large numbers of sub-microscopic parasitaemia in this study was supported by meta-analysis that demonstrates high proportion of sub-microscopic infection of *P. falciparum* in areas of low transmission [26], indicating that those with little previous exposure are able to control parasite densities. The presence of high proportion of sub-microscopic infections could indicate a recent decrease in transmission in Ethiopia and this is consistent with data from northern Tanzania showing high (33%) prevalence of almost entirely sub-microscopic infections during a time of declining transmission [7]. In a study conducted in Cambodia, microscopy detected a total of 350 *P. falciparum* infections while PCR detected a further 331 *P. falciparum* infections from microscopy-negative samples [32]. The importance of sub-microscopic parasitaemia in sustaining the parasite population has been indicated [33]. PCR also has a limit of detection; the number of sub-microscopic infections is likely higher than reported in this study.

The prevalence of microscopic *P. falciparum* infection decreases with age since younger age groups have the highest incidences as determined by microscopy, RDT and PCR. On the other hand, the proportion of sub-microscopic *P. falciparum* infections was higher in older age groups. This is because increasing age has been clearly linked to lower parasite densities [34] and most infections in older children and adults are sub-microscopic compared with young children [7]. Children present with symptomatic malaria at a younger age in areas of high transmission than in areas with lower transmission [17]. Under conditions of very low transmission, the risk of clinical disease extends into adulthood [35] where risks of a clinical event are more directly related to the risks of infection than the effect of acquired clinical immunity. But an increase in the prevalence of sub-microscopic *P. falciparum* infection with age may explain the fact that infections will be controlled and remain asymptomatic in older age groups because clinical immunity develops overtime.

The observation of high prevalence of PCR-based sub-microscopic *P. falciparum* in the study area has important implications for malaria control measures in Ethiopia since such infections are important contributors to the infectious reservoir [36-38], because, even at low densities, these infections could be a potential source of transmission for vectors [26,33] and a potential source of malaria attack within the population. Several studies have indicated that the contribution of sub-microscopic parasites to malaria transmission in individuals was similar to those individuals having microscopic gametocytes [38] while others indicated that sub-microscopic carriers were several times less infectious than the microscopic carriers [7]. For assessing progress in reducing malaria transmission, PCR seems the best tool for the estimation of parasite prevalence in the general population.

In this study, older age was associated with increased sub-microscopic carriages. This finding is in agreement with a study by Manjurano and his colleagues [37] where individuals older than 15 years were three times more likely to have sub-microscopic parasites than younger individuals. It is apparent that the risk of malarial infection could be reduced by bed net use. In this study, use of bed nets was not found to be protective against sub-microscopic carriage. This is supported by a study where 50% of net users from high transmission areas were PCR positive compared to 75% of non net users at the lowest altitude [37]. The high incidences of sub-microscopic carriages observed in older age groups as compared to the younger age group (<15 years) in this study is supported by another study [39] conducted in Uganda. Moreover, Vafa and her colleagues [40] have reported that sub-microscopic carriage differs in relation to transmission intensity and age. Nevertheless, asymptomatic carriage is probably a common occurrence in the study area. Further studies to unveil the magnitude of sub-microscopic asymptomatic carriage will be indispensable for guiding and monitoring future elimination efforts in Ethiopia.

## Conclusions

Large numbers of sub-microscopic *P. falciparum* infections were identified in the study area. Even though malaria transmission is seasonal and unstable in the area, significant numbers of *Plasmodium*-infected individuals were asymptomatic and carry sub-microscopic parasite densities below the threshold of microscopy and RDT. It is important to maximize detection of cases (symptomatic and sub-microscopic asymptomatic) presumably infectious to mosquitoes using enhanced diagnostic techniques. A molecular test (PCR) is more sensitive in detecting low levels of sub-microscopic *P. falciparum* infections and prevalence determination of malaria in a given population. Thus, PCR may be the best tool for measuring *Plasmodium* prevalence than microscopy and RDT. The presence of sub-microscopic *P. falciparum* infections makes control and reduction of malaria transmission easier said than done since they may not be detected by the conventional diagnostic methods, i.e. microscopy or RDTs. Sub-patent malaria infections, detectable only by molecular techniques (PCR), can be relatively common in areas of low malaria endemicity in Ethiopia and these may substantially contribute to maintaining malaria transmission in the country. The role of sub-microscopic parasite carriages in human-mosquito transmission need to be determined especially when malaria control and elimination is prioritized.

## Competing interests

The authors declare that they have no competing interests.

## Authors’ contributions

LG, BE, AA and GS designed the study and involved in all stages of this study. LG was responsible for sample collection and prepared the draft manuscript. BE and AA coordinated the field work. GS, NE and LG extracted the DNA and did the molecular analysis. All authors contributed to interpretation of data, writing and revising the manuscript, and have seen and approved the final version.

## References

[B1] Abeku TA Kristan M Jones C Beard J Mueller DH Okia M Rapuoda B Greenwood B Cox J Determinants of the accuracy of rapid diagnostic tests in malaria case management: evidence from low and moderate transmission settings in the East African highlands Malar J 2008 7 202 10.1186/1475-2875-7-202 18834523 PMC2571107

[B2] Alves FP Durlacher RR Menezes MJ Krieger H Silva LH Camargo EP High prevalence of asymptomatic *Plasmodium vivax* and *Plasmodium falciparum* infections in native Amazonian populations Am J Trop Med Hyg 2002 66 641 648 12224567 10.4269/ajtmh.2002.66.641

[B3] Baird JK Basri H Weina P MaGuire JD Barcus MJ Picarema H Elyazar IR Ayomi ES Adult Javanese migrants to Indonesian Papua at high risk of severe disease caused by malaria Epidemiol Infect 2003 131 791 797 10.1017/S0950268803008422 12948380 PMC2870021

[B4] Bisoffi Z Sirima S Menten J Pattaro C Angheben A Gobbi F Tinto H Lodesani C Neya B Gobbo M Vanden Ende J Accuracy of a rapid diagnostic test on the diagnosis of malaria infection and of malaria-attributable fever during low and high transmission season in BurkinaFaso Malar J 2010 9 192 10.1186/1475-2875-9-192 20609211 PMC2914059

[B5] Biswas S Tomar D Rao DN Investigation of the kinetics of histidine-rich protein 2 and of the antibody responses to this antigen, in a group of malaria patients from India Ann Trop Med Parasitol 2005 99 553 562 10.1179/136485905X51463 16156968

[B6] Brenner H Gefeller O Variation of sensitivity, specificity, likelihood ratios and predictive values with disease prevalence Stat Med 1997 16 981 991 10.1002/(SICI)1097-0258(19970515)16:9<981::AID-SIM510>3.0.CO;2-N 9160493

[B7] Okell LC Bousema T Griffin JT Ouédraogo AL Ghani AC Drakeley CJ Factors determining the occurrence of sub-microscopic malaria infections and their relevance for control Nat Commun 2012 3 1237 23212366 10.1038/ncomms2241 PMC3535331

[B8] Endeshaw T Gebre T Ngondi J Graves PM Shargie EB Ejigsemahu Y Ayele B Yohannes G Teferi T Messele A Zerihun M Genet A Mosher AW Emerson PM Richards FO Evaluation of light microscopy and rapid diagnostic test for the detection of malaria under operational field conditions: a household survey in Ethiopia Malar J 2008 7 118 10.1186/1475-2875-7-118 18598344 PMC2474640

[B9] Houwen B Blood film preparation and staining procedures Clin Lab Med 2002 22 1 14 10.1016/S0272-2712(03)00064-7 11933570

[B10] Gamboa D Ho MF Bendezu J Torres K Chiodini PL Barnwell JW Incar-dona S Perkins M Bell D Mc Carthy J Cheng Q A large proportion of P. falciparum isolates in the Amazon region of Peru lack pfhrp2 and pfhrp3: implications for malaria rapid diagnostic tests PLoS One 2010 5 e8091 10.1371/journal.pone.0008091 20111602 PMC2810332

[B11] Coleman RE Kumpitak C Ponlawat A Maneechai N Phunkitchar V Rachapaew N Zollner G Sattabongkot J Infectivity of asymptomatic *Plasmodium*-infected human populations to Anopheles dirus mosquitoes in western Thailand J Med Entomol 2004 41 201 208 10.1603/0022-2585-41.2.201 15061279

[B12] Graz B Willcox M Szeless T Rougemont A “Test and treat” or presumptive treatment for malaria in high transmission situations? A reflection on the latest WHO guidelines Malar J 2011 10 136 10.1186/1475-2875-10-136 21599880 PMC3123602

[B13] Harris I Sharrock WW Bain LM Gray KA Bobogare A Boaz L Lilley K Krause D Vallely A Johnson ML Gatton ML Shanks GD Cheng Q A large proportion of asymptomatic *Plasmodium* infections with low and sub-microscopic parasite densities in the low transmission setting of Temotu Province, Solomon Islands: challenges for malaria diagnostics in an elimination setting Malar J 2010 9 254 10.1186/1475-2875-9-254 20822506 PMC2944341

[B14] McKenzie FE Prudhomme WA Magill AJ Forney JR Permpanich B Lucas C Gasser RA Jr Wongsrichanalai C White blood cell counts and malaria J Infect Dis 2005 192 323 330 10.1086/431152 15962228 PMC2481386

[B15] Plowe C Djimde A Bouare M Doumbo O Wellems TE Pyrimethamine and proguanil resistance-conferring mutations in *Plasmodium falciparum* dihydrofolate reductase: polymerase chain reaction methods for surveillance in Africa Am J Trop Med Hyg 1995 52 565 568 7611566 10.4269/ajtmh.1995.52.565

[B16] Masanja MI McMorrow M Kahigwa E Kachur SP McElroy PD Health workers’ use of malaria rapid diagnostic tests (RDTs) to guide clinical decision making in rural dispensaries, Tanzania Am J Trop Med Hyg 2010 83 1238 1241 10.4269/ajtmh.2010.10-0194 21118927 PMC2990037

[B17] Serwanga A Harris JC Kigozi R Menon M Bukirwa H Gasasira A Kakeeto S Kizito F Quinto E Rubahika D Nasr S Filler S Kamya MR Dorsey G Improved malaria case management through the implementation of a health facility-based sentinel site surveillance system in Uganda PLoS One 2011 6 e16316 10.1371/journal.pone.0016316 21283815 PMC3023768

[B18] Msellem MI Martensson A Rotllant G Bhattarai A Stromberg J Kahigwa E Garcia M Petzold M Olumese P Ali A Bjorkman A Influence of rapid malaria diagnostic tests on treatment and health outcome in fever patients, Zanzibar: a crossover validation study PLoS Med 2009 6 e1000070 10.1371/journal.pmed.1000070 19399156 PMC2667629

[B19] Reyburn H Mbakilwa H Mwangi R Mwerinde O Olomi R Drakeley C Whitty CJ Rapid diagnostic tests compared with malaria microscopy for guiding outpatient treatment of febrile illness in Tanzania: randomised trial BMJ 2007 334 403 10.1136/bmj.39073.496829.AE 17259188 PMC1804187

[B20] Perneger TV Szeless T Rougemont A Utility of the detection of *Plasmodium* parasites for the diagnosis of malaria in endemic areas BMC Infect Dis 2006 6 81 10.1186/1471-2334-6-81 16670024 PMC1475866

[B21] Cheng A Bell D Evidence behind the WHO guidelines: hospital care for children: what is the precision of rapid diagnostic tests for malaria? J Trop Pediatr 2006 52 386 389 10.1093/tropej/fml037 16943212

[B22] WHO, FIND, CDC, TDR Malaria rapid diagnostic test performance: results of WHO product testing malaria RDTs: round 2 2009 Geneva: World Health Organization

[B23] Willcox ML Sanogo F Graz B Forster M Dakouo F Sidibe O Falquet J Giani S Diakite C Diallo D Rapid diagnostic tests for the home-based management of malaria, in a high-transmission area Ann Trol Med Parasitol 2009 103 3 16 10.1179/136485909X384983 19173772

[B24] Ochola LB Vounatsou P Smith T Mabaso M Newton C The reliability of diagnostic techniques in the diagnosis and management of malaria in the absence of a gold standard Lancet Infect Dis 2006 6 582 588 10.1016/S1473-3099(06)70579-5 16931409

[B25] Steenkeste N Rogers WO Okell L Jeanne I Incardona S Duval L Chy S Hewitt S Chou M Socheat D Babin FX Ariey F Rogier C Sub-microscopic malaria cases and mixed malaria infection in a remote area of high malaria endemicity in Rattanakiri province, Cambodia: implication for malaria elimination Malar J 2010 9 108 10.1186/1475-2875-9-108 20409349 PMC2868861

[B26] Okell LC Ghani AC Lyons E Drakeley CJ Infection in *Plasmodium falciparum*–endemic populations: a systematic review and meta-analysis J Infect Dis 2009 200 1509 1517 10.1086/644781 19848588

[B27] da Silva-Nunes M Moreno M Conn Jan E Gamboa D Abeles S Vinetz JM Ferreira MU Amazonian malaria: Asymptomatic human reservoirs, diagnostic challenges, environmentally driven changes in mosquito vector populations, and the mandate for sustainable control strategies Acta Trop 2012 121 281 291 10.1016/j.actatropica.2011.10.001 22015425 PMC3308722

[B28] Tjitra E Suprianto S McBroom J Currie BJ Anstey NM Persistent ICT malaria P.f/P.v panmalarial and HRP2 antigen reactivity after treatment of *Plasmodium falciparum* malaria is associated with gametocytemia and results in false-positive diagnoses of *Plasmodium vivax* in convalescence J Clin Microbiol 2001 39 1025 1031 10.1128/JCM.39.3.1025-1031.2001 11230422 PMC87868

[B29] Swarthout TD Counihan H Senga RK van den Broek I Swarthout TD Counihan H Senga RKK van den Broek I Paracheck-Pf accuracy and recently treated *Plasmodium falciparum* infections: is there a risk of over-diagnosis? Malar J 2007 6 58 10.1186/1475-2875-6-58 17506881 PMC1890550

[B30] Zurovac D Midia B Ochola SA English M Snow RW Microscopy and outpatient malaria case management among older children and adults in Kenya Trop Med Int Health 2006 11 432 440 10.1111/j.1365-3156.2006.01587.x 16553926

[B31] Keating J Miller JM Bennett A Moonga HB Eisele TP *Plasmodium falciparum* parasite infection prevalence from a household survey in Zambia using microscopy and a rapid diagnostic test: implications for monitoring and evaluation Acta Trop 2009 112 277 282 10.1016/j.actatropica.2009.08.011 19682968

[B32] Pichon G Awono-Ambene HP Robert V High heterogeneity in the number of *Plasmodium falciparum* gametocytes in the bloodmeal of mosquitoes fed on the same host Parasitology 2000 121 115 120 10.1017/S0031182099006277 11085230

[B33] Sama W Owusu-Agyei S Felger I Dietz K Smith T Age and seasonal variation in the transition rates and detectability of *Plasmodium falciparum* malaria Parasitology 2006 13 13 21 16393349 10.1017/S0031182005008607

[B34] Reyburn H Mbatia R Drakeley C Bruce J Carneiro I Olomi R Cox J Nkya W Lemnge M Greenwood BM Riley EM Association of transmission intensity and age with clinical manifestations and case fatality of severe *Plasmodium falciparum* malaria JAMA 2005 293 1461 1470 10.1001/jama.293.12.1461 15784869

[B35] Snounou G Pinheiro L Gonçalves A Fonseca L Dias F Brown KN do Rosario VE The importance of sensitive detection of malaria parasites in the human and insect hosts in epidemiological studies, as shown by the analysis of field samples from Guinea Bissau Trans R Soc Trop Med Hyg 1993 87 649 653 10.1016/0035-9203(93)90274-T 8296364

[B36] Diallo A Ndam NT Moussiliou A Dos Santos S Ndonky A Borderon M Oliveau S Lalou R Le Hesran JY Asymptomatic carriage of *Plasmodium* in urban Dakar: the risk of malaria should not be underestimated PLoS One 2012 7 e31100 10.1371/journal.pone.0031100 22363558 PMC3283586

[B37] Manjurano A Okell L Lukindo T Reyburn H Olomi R Roper C Clark TG Joseph S Riley EM Drakeley C Association of sub-microscopic malaria parasite carriage with transmission intensity in northeastern Tanzania Malar J 2011 10 370 10.1186/1475-2875-10-370 22177014 PMC3276450

[B38] Schneider P Bousema JT Gouagna LC Otieno S Van de Vegte-Bolmer M Omar SA Sauerwein RW Sub-microscopic *Plasmodium falciparum* gametocyte densities frequently result in mosquito infection Am J Trop Med Hyg 2007 76 470 474 17360869

[B39] Proietti C Pettinato DD Kanoi BN Ntege E Crisanti A Riley EM Egwang TG Drakeley C Bousema T Continuing intense malaria transmission in northern Uganda Am J Trop Med Hyg 2011 84 830 837 10.4269/ajtmh.2011.10-0498 21540398 PMC3083756

[B40] Vafa M Troye-Blomberg M Anchang J Garcia A Migot-Nabias F Multiplicity of *Plasmodium falciparum* infection in asymptomatic children in Senegal: relation to transmission, age and erythrocyte variants Malar J 2008 7 17 10.1186/1475-2875-7-17 18215251 PMC2267475

